# Neutrophil Extracellular Traps Serve as Key Effector Molecules in the Protection Against *Phialophora verrucosa*

**DOI:** 10.1007/s11046-021-00554-0

**Published:** 2021-05-19

**Authors:** Qin Liu, Wenjuan Yi, Si Jiang, Jiquan Song, Pin Liang

**Affiliations:** Department of Dermatology, Zhongnan Hospital of Wuhan University, Wuhan, Hubei China

**Keywords:** *Phialophora verrucosa*, Neutrophils, Neutrophil extracellular traps, Fungi killing

## Abstract

*Phialophora verrucosa* (*P. verrucosa*) is a pathogen that can cause chromoblastomycosis and phaeohyphomycosis. Recent evidence suggests that neutrophils can produce neutrophil extracellular traps (NETs) that can protect against invasive pathogens. As such, we herein explored the in vitro functional importance of *P. verrucosa-*induced NET formation. By assessing the co-localization of neutrophil elastase and DNA, we were able to confirm the formation of classical NETs entrapping *P. verrucosa* specimens. Sytox Green was then used to stain these NETs following neutrophil infection with *P. verrucosa* in order to quantify the formation of these extracellular structures. NET formation was induced upon neutrophil exposure to both live, UV-inactivated, and dead *P. verrucosa* fungi. The ability of these NETs to kill fungal hyphae and conidia was demonstrated through MTT and pouring plate assays, respectively. Overall, our results confirmed that *P. verrucosa* was able to trigger the production of NETs, suggesting that these extracellular structures may represent an important innate immune effector mechanism controlling physiological responses to *P. verrucosa* infection, thereby aiding in pathogen control during the acute phases of infection.

## Introduction

*Phialophora verrucosa* (*P. verrucosa*) is a species of dematiaceous fungi that is often found in soil, wood, and decomposing vegetation [1]. In humans, infection with this fungus can cause chromoblastomycosis and phaeohyphomycosis, with severe *P. verrucosa* infections most often occurring in immunosuppressed individuals [1, 2]. Patients suffering from these infections generally exhibit a chronic disease with a prolonged, refractory course that adversely affects their quality of life and can potentially result in mortality [3].

At present, the immunological factors important for the control of *P. verrucosa* infections remain poorly understood. Polymorphonuclear neutrophils (PMNs) are circulating phagocytic cells in humans, wherein they function as short-lived effectors cells that can rapidly respond to invasive microorganisms [4], neutralizing them via phagocytosis, respiratory burst activity, and the release of cytotoxic compounds and structures [4, 5]. We have previously demonstrated that PMNs can readily phagocytose and thereby kill *P. verrucosa* [3], indicating that these cells may play an important role in host innate immune responses to this fungus.

Many recent studies have highlighted the ability of PMNs to kill extracellular pathogens via the release of fibrous neutrophil extracellular traps (NETs), which can entrap and kill fungi and bacteria without direct phagocytic uptake [6]. These NETs are made up of a network of DNA embedded with histones, neutrophil elastase (NE), and myeloperoxidase (MPO) [6–10] and play vital roles in the clearance of certain viruses, bacteria, fungi, and parasites [6, 8, 11–14], including *Staphylococcus aureus, Salmonella typhimurium* [6, 15], *Escherichia coli* [15], Streptococcus [16, 17], *Listeria monocytogenes* [18], *Besnoitia besnoiti* [14], and *Candida albicans* [8]. PMNs have also been reported to produce NETs upon exposure to *Aspergillus fumigatus* and *Cryptococcus neoformant* [19, 20]. However, no data are currently available pertaining to the roles of NETs in the context of dematiaceous fungal infections.

The present study was therefore designed to establish whether *P. verrucosa-*induced NET formation is an important effector mechanism governing the ability of PMNs to kill these fungi during the acute phase of infection.

## Materials and Methods

### Ethics Statement

The ethics committee of the Zhongnan Hospital of Wuhan University approved this study, which was consistent with the Declaration of Helsinki. All patients provided written informed consent to participate, and PMNs were isolated from samples of venous blood obtained from healthy volunteer donors.

### Fungal Culture and Preparation

For this study, a clinical *P. verrucosa* isolate from a consenting patient (NO.WPMC060039) was utilized. Conidia were obtained by culturing fungi for 2 weeks on potato dextrose agar (PDA) at 28 °C, after which PBS was used to flood the surface of this culture. A hemocytometer was then utilized to count these conidia, and hyphae were prepared by incubating 10^5^ conidia in liquid RPMI medium for 5 days at 28 °C. Conidial opsonization was achieved by combining a suspension of these conidia with 10% [v/v] pooled serum for 15 min at 37 °C. Samples were then spun down and washed two times prior to resuspension in PBS [3].

### Neutrophil Isolation

PMN isolation was conducted as reported previously [3]. Briefly, a PolymorphPrep kit (Axis-Shield, Norway) was used for differential centrifugation, after which red blood cells were lysed and remaining granulocytes were resuspended in RPMI-1640 (HyClone, USA) containing 10% FBS (HyClone, USA), penicillin/streptomycin, and 2 mM l-glutamine (Sigma-Aldrich, USA). PMNs were incubated at 37 °C in a 5% CO2 incubator before use.

### NET Visualization and Staining

As discussed in prior studies [21], human PMNs were treated with PMA (100 nM) or *P. verrucosa* conidia that either had or had not been opsonized (MOI = 10) for 4 h on poly-l-lysine-treated coverslips, after which 4% paraformaldehyde (Merck) was used to fix samples. Samples were then washed thrice with PBS, blocked with 1% BSA (Invitrogen), and stained with anti-NE (Abcam) for 24 h at room temperature. After three washes, samples were mounted in anti-fading buffer, and DNA counterstaining was conducted by exposing coverslips to DAPI at room temperature for 10 min. An inverted fluorescence microscope (Leica) was then used for sample visualization.

### NET Quantification

SYTOX Green (Invitrogen) was used to assess NET formation. Human PMNs were combined with *P. verrucosa* conidia at a 10:1 ratio with or without serum for 1–3 h at 37 °C. To assess the impact of conidia viability of NET production, conidia were either attenuated via ultraviolet (UV) light exposure as detailed previously [22] (60 min, 230 V/50–60 Hz) or were heat-inactivated (100 °C, 30 min). As a positive control, PMNs were stimulated with PMA (Sigma; 25 nM). To assess the dose-dependency of NET induction, PMNs were treated at a range of PMN:conidia ratios (1:5, 1:10, or 1:20 for 2 h). Maximum extracellular DNA levels were estimated by lysing PMNs with Triton X-100 (Sigma). NET formation was blocked via treatment with DNase I (Invitrogen; 45 U). Samples were then spun down, and supernatants were transferred to 96-well plates (100 µL/well), after which 30 μL of SYTOX Green (10 × working stock; 50 μM) was added per well. A fluorescence microplate reader was then used to monitor NET formation based on fluorescent signal. Percentage NET formation was then determined by subtracting background fluorescence (determined following DNase addition) and dividing by the maximum fluorescent signal observed for lysed PMNs incubated with Triton X-100.

### Assessment of NET-Mediated *P. verrucosa* Killing

All assays were conducted as in prior reports [8]. Briefly, human PMNs (2 × 10^6^/mL) were added to tissue culture plates in RPMI-1640 with or without cytochalasin D (10 μg/mL) for 20 min, after which conidia that were or were not opsonized were added for 1–3 h at a PMN:conidia ratio of 10:1. Cytochalasin D (Sigma) blocked phagocytosis without affecting NET formation.

To assess the functional importance of NETs as mediators of extracellular fungal killing, samples were pre-treated with protease-free DNase-1 (40 U/mL, Worthington) to facilitate NET degradation prior to *P. verrucosa* conidia addition as above. At 2 h post-infection, numbers of viable conidia were determined via a pour plate approach using PDA plate, with *P. verrucosa* colony forming units (CFUs) being counted following a 6-day incubation. The percentage killing was then determined based on comparisons with control samples in which conidia were incubated without any PMNs. All analyses were conducted in triplicate.

A 3-(4,5-dimethylthiazol-2-yl)-2,5-diphenyltetrazolium bromide (MTT)-based colorimetric approach was used to assess hyphal damage, as discussed in prior studies [3, 23]. *P. verrucosa* hyphae were prepared by incubating 10^5^ conidia in liquid RPMI for 5 days at 28 °C. PMNs were then combined with these hyphal preparations for 2 or 4 h at 37 °C (MOI = 10) in the presence or absence of cytochalasin D (10 μg/mL). Samples were then lysed with a water/NaOH solution (pH = 11), washed in PBS, and for an additional 3 h with the MTT reagent (Invitrogen). Hydrogenases within functional hyphae are able to cleave MTT to yield a purple MTT-formazan precipitate that can then be dissolved with isopropanol and quantified with a microplate spectrophotometer (Bio-Tek Instruments, VT, USA) at 570 nm. The killing of hyphae (%) was determined as follows: killing (%) = [1-(A570 of fungi incubated with cells-A570 of cells alone)/A570 of fungi alone] × 100.

### Statistical Analysis

Figures were prepared by using GraphPad Prism 5.0, and statistical analysis was performed using SPSS 16.0. Data are given as means ± SEM and were compared by ANOVAs. Experiments were repeated in triplicate. Differences were considered to be statistically significant at *P* values of < 0.05.

## Results

### *Phialophora verrucosa* Induces NET Formation

**Fig. 1:**
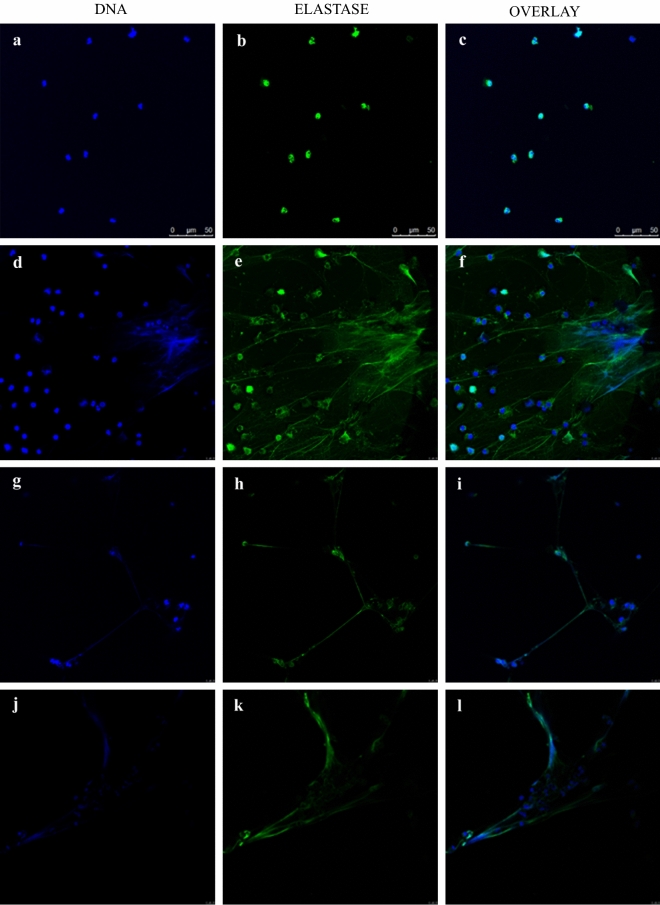
Visualization of neutrophil-derived NETs. PMNs at rest (panels **a**, **b**, **c**) or that had been stimulated with PMA (100 nM, 4 h at 37 °C) (panels **d**, **e**, **f**), non-opsonized *P. verrucosa* (panels **g**, **h**, **i**), and opsonized *P. verrucosa* (panels **j**, **k**, **l**) were fixed and DNA staining was performed (DAPI; panels **a**, **d**, **g**, **j**), as was NE staining (panels **b**, **e**, **h**, **k**). DNA and NE signal overlays are shown in panels **c**, **f**, **i**, and **l** for these four respective treatments. Scale bar = 50 μm

To assess the ability of *P. verrucosa* to induce NET production by human PMNs, we exposed these cells for 4 h to PMA (100 nM) or to *P. verrucosa* conidia that either had or had not been opsonized (MOI = 10) (Fig. 1). Samples were then fixed and stained for NE (green) and DNA (blue), with NETs being observed as fibrous blue structures containing green NE granules. While NET production from unstimulated PMNs was minimal (Fig. 1c), PMA treatment resulted in the marked enhancement of NET formation (Fig. 1f). Extended exposure to opsonized conidia resulted in more robust NET formation than did non-opsonized conidia exposure, although in both cases the production was less than that observed in response to PMA stimulation. Together, these results indicated that *P. verrucosa* was able to induce NET generation by human PMNs, with opsonization of these fungi enhancing such antimicrobial structure formation.

### NET Quantification

**Fig. 2:**
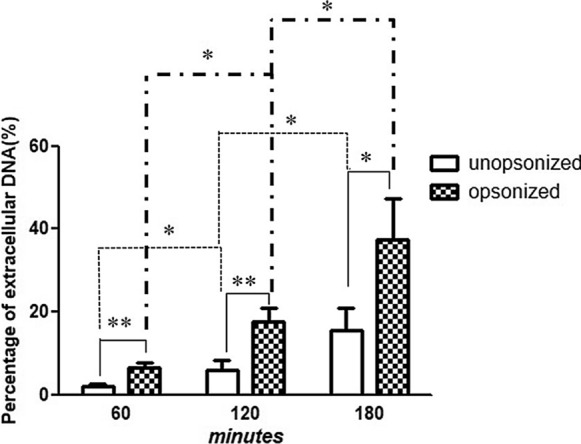
Conidia-induced NET formation kinetics. PMNs were combined with *P. verrucosa* conidia that had or had not been opsonized at a 1:10 PMN:conidia ratio for the indicated time periods, after which SYTOX Green fluorescence intensity was used to assess levels of extracellular DNA in these samples. Samples were analyzed in triplicate, and *P* < 0.05 was the significance threshold

**Fig. 3:**
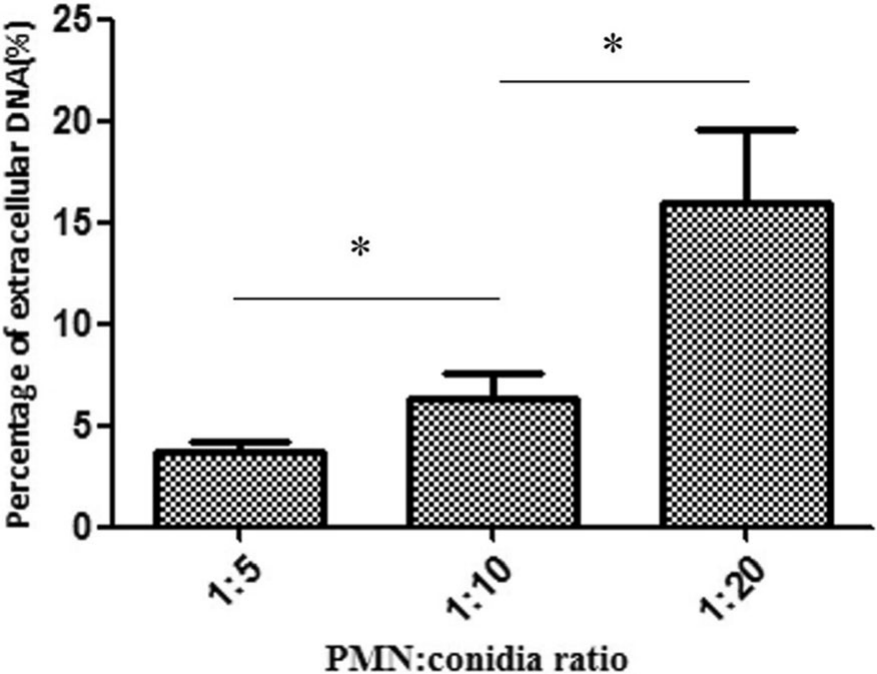
Conidia trigger NET formation in a dose-dependent fashion. PMNs and *P. verrucosa* conidia were incubated at different ratios (PMN:conidia = 1:5, 1:10, 1:20). SYTOX Green fluorescence intensity was then used to assess levels of extracellular DNA in these samples. *P* < 0.05 was the significance threshold

We next explored the impact of opsonization on *P. verrucosa* conidia-induced NET induction in a quantitative manner, revealing that exposure to opsonized NETs was associated with a significant increase in NET formation relative to exposure to non-opsonized NETs (Fig. 2) (*, *P* < 0.05; **, *P* < 0.01), suggesting that serum opsonization can facilitate NET production. We also found that NET formation was induced in a time-dependent manner upon exposure to both opsonized and non-opsonized conidia over a 1–3 h period, with a significant difference between time points (Fig. 2) (*P* < 0.05). Such *P. verrucosa-*induced NET formation was also dose dependent (Fig. 3) (*, *P* < 0.05; **, *P* < 0.01).

The induction of NET formation in response to *P. verrucosa* conidia is not dependent upon fungal viability.

**Fig. 4:**
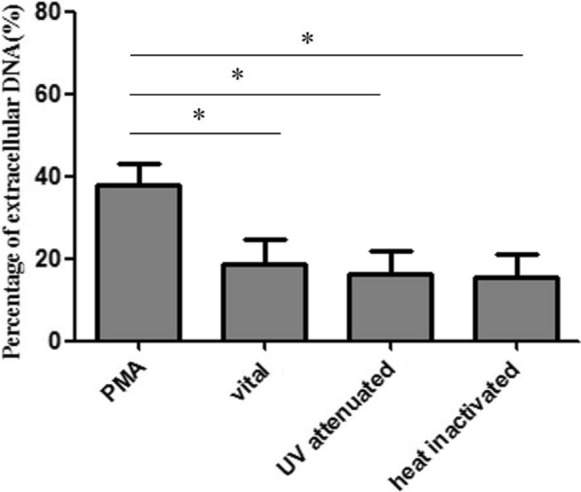
*P. verrucosa* conidia induce NET formation. Human PMNs were exposed to viable, UV-inactivated, and heat-inactivated *P. verrucosa* conidia (MOI = 10) for 3 h. PMA stimulation (25 nM) was utilized as a positive control. SYTOX Green fluorescence intensity was used to assess levels of extracellular DNA in these samples. *P* < 0.05 was the significance threshold

We next evaluated the relationship between *P. verrucosa* viability and NET formation, revealing that viable, UV-attenuated, and non-viable conidia were all able to induce similar levels of NET formation that were reduced relative to those induced by PMA stimulation (Fig. 4) (*P* < 0.05). Overall, these data demonstrated that *P. verrucosa* conidia-induced NET formation in a manner that was not dependent upon conidial viability.

### Assessment of NET-Mediated Killing of Hyphae and Conidia

As *P. verrucosa* was able to induce NET formation, we next evaluated the ability of these NETs to kill *P. verrucosa* hyphae and conidia. To specifically evaluate extracellular killing, PMNs were treated with the actin inhibitor cytochalasin D to suppress phagocytosis. Plating and MTT assays were used to assess fungal survival at the indicated time points.

**Fig. 5:**
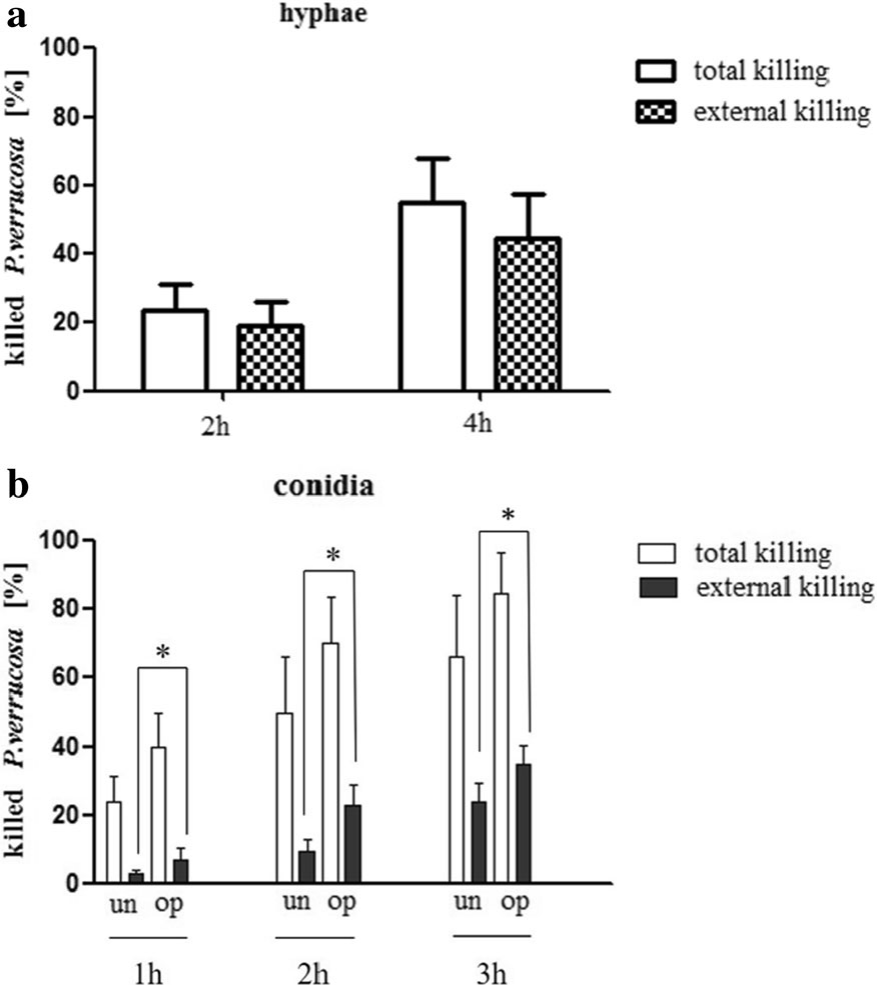
NETs can entrap and kill *P. verrucosa* hyphae and conidia. The hyphae (**a**) or conidia (**b**) of *P. verrucosa* were used to infect PMNs for the indicated periods time, after which *P. verrucosa* conidial colony-forming units were quantified and the hyphae killing were tested by MTT-based colorimetric approach. Extracellular (external) killing was assessed in the presence of the actin inhibitor cytochalasin D, while total killing was assessed in the absence of this compound. *P* < 0.05 was the significance threshold

Hyphal and conidial killing in these experimental settings is shown in Fig. 5, with the percentage of killing being assessed relative to control samples. Total killing was assessed by combining *P. verrucosa* and PMNs in the absence of cytochalasin D, reflecting both extracellular killing and phagocytic killing. Extracellular killing was assessed based on the percentage of killing in samples treated with cytochalasin D in which phagocytosis was blocked.

We found that the duration of treatment was associated with a significant increase in both overall and extracellular PMN-mediated killing of *P. verrucosa* spores and mycelia (*P* < 0.05). At 2 and 4 h post-treatment of PMNs with hyphae, 20% and 40–50% of these hyphae, respectively, were killed in the presence of cytochalasin D as a metric for extracellular (external) killing (Fig. 5a). When no cytochalasin D was added, overall *P. verrucosa* hyphae killing ranged from 20–25% and 50–60% at these respective time points, with no significant differences in the total and extracellular PMN-mediated killing of these mycelia at these individual time points (*P* > 0.05).

To better understand the role of serum-mediated opsonization in the context of NET-induced *P. verrucosa* killing, PMNs were combined with serum-opsonized conidia. The results of this experiment suggested that PMN-mediated extracellular killing of conidia was enhanced for opsonized conidia relative to non-opsonized conidia (Fig. 5b) (*P* < 0.05).

**Fig. 6:**
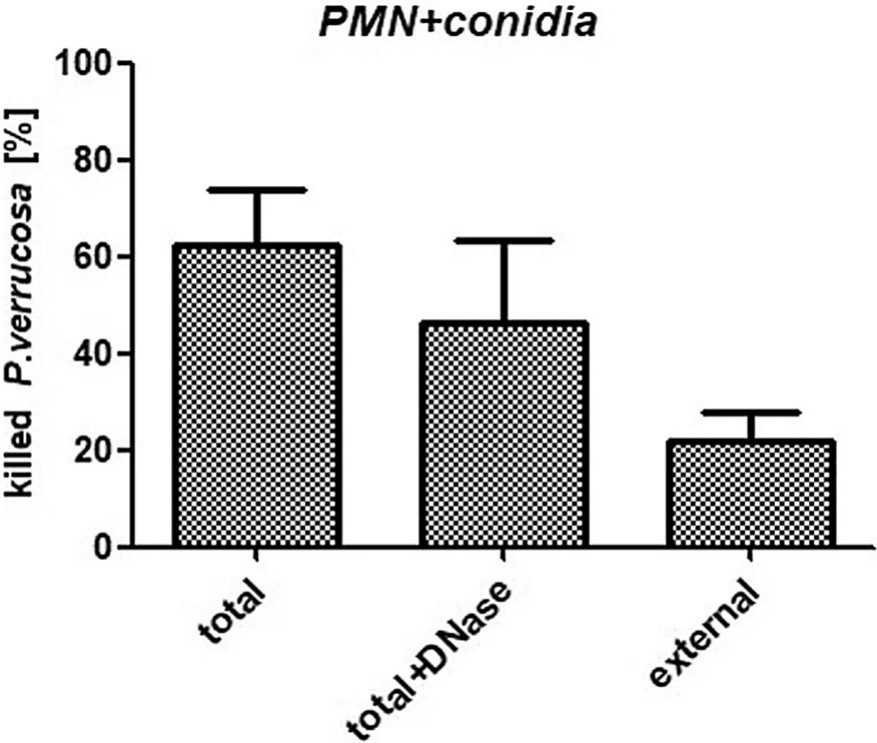
NETs mediate the extracellular killing of *P. verrucosa* conidia. DNase I (40 U/mL) was used to digest NETs, and *P. verrucosa* was used to infect PMNs. Such DNase-I treatment was associated with a 16% reduction in fungal killing (from 62 to 46%), with this reduction being similar to the observed extracellular killing activity (21%). Such extracellular (external) killing was assessed in the presence of the actin inhibitor cytochalasin D, while total killing was assessed in the absence of this compound

As NETs are composed of a DNA scaffold, we treated samples with protease-free DNase-1 at time of infection to degrade these structures [6]. Samples were not washed following treatment such that DNase remained present in the solution. The degree of PMN-mediated *P. verrucosa* killing was reduced from 62 to 46% following DNase treatment (Fig. 6). The degree of this reduction (16%) was similar to the observed degree of extracellular killing (21%), suggesting that NETs were the primary mediators of extracellular fungal killing consistent with prior reports [8].

## Discussion

Neutrophils are important short-lived effector cells that play essential roles in coordinating innate immune responses against pathogens. The production of NETs is one of the primary mechanisms whereby these PMNs can combat extracellular pathogens [24], and these NETs are vital for non-specific prevention of the spread of such pathogens [8, 25].

Following microbe-mediated activation, neutrophils are recruited to infected tissues wherein they generate an oxidative burst [26]. Both opsonized and non-opsonized *P. verrucosa* conidia were able to activate PMNs and trigger the production of NETs (Fig. 1). Quantitative analyses revealed that NET formation was induced in a time-dependent manner over a 1–3 h period after exposure to these conidia, with opsonized conidia inducing more robust NET formation relative to non-opsonized conidia at all analyzed time points (Fig. 2; *P* < 0.05). This suggests that opsonization can enhance NET formation, although we cannot exclude the possibility that multiple parallel signaling pathways may govern NET formation in this context [8]. It is speculated that opsonins in serum might play an crucial role in the improvement in NETs-formation. Previous studies have reported that opsonins, such as immunoglobulins and complement proteins, participated in the killing and elimination of pathogenic microorganisms by interacting with the corresponding receptors on the surface of leukocyte, including FcγR and CR3 [23, 27–31]. Therefore, the triggering of NET production in response to opsonized *P. verrucosa* may be mediated by complement or Fcγ receptors, whereas for non-opsonized conidia these responses may be regulated by unopsonized pathogens pattern recognition receptors (PRRs) including Toll-like receptors [32] or dectin [33]. Additionally, we found that NETs could be triggered at 1 h after exposure to unopsonized as well as opsonized conidia, inconsistent with previous report that naive neutrophils did not form NETs by *C. albicans* stimulation within 1 h [8]. It is possible that different signaling pathways might exist for NET-triggering among populations.

Since first being detected as an innate immune effector mechanism [6], NETs have been shown to be important mediators of responses to fungal and bacterial pathogens [6, 8, 11–20]. Herein, we found that *P. verrucosa* was able to trigger NET formation as detected via immunofluorescent imaging. Quantitative assays further indicated that *P. verrucosa* was able to trigger NET formation in a dose-dependent fashion (Fig. 3). We additionally evaluated the impact of *P. verrucosa* viability on conidia-induced NET formation (Fig. 4), but we observed no significant differences in NET production when PMNs were stimulated with viable, UV- attenuated, or heat-inactivated conidia, similar to what has been reported for Apicomplexan Parasite *Besnoitia besnoiti* [14].

We have previously shown that PMNs can phagocytose and kill *P. verrucosa* conidia and hyphae [3]. Herein, we further showed that NETs also facilitated PMN-mediated *P. verrucosa* killing. And the observed killing of *P. verrucosa* conidia occurred primarily intracellularly (Fig. 5b), whereas extracellular killing was the primary mechanism of *P. verrucosa* hyphae clearance (Fig. 5a). As we found that DNase treatment was sufficient to reduce PMN-mediated killing of *P. verrucosa* by a similar percentage to the observed percentage of extracellular killing (16% vs. 21%, respectively) (Fig. 6), this suggested that NETs were the primary drivers of such extracellular killing. NETs are thus likely to be essential to the efficient killing of *P. verrucosa* by these innate immune cells.

Neutrophils rapidly and continuously migrate to infected tissues and are activated by specific cytokines and pathogen-related signaling mechanisms. Neutrophil-derived NETs may represent a key mechanism whereby these cells are able to kill *P. verrucosa* [8]*,* in addition to the phagocytic activity that we have demonstrated previously [3]. NETs may be particularly important as a means of killing microbes that are too large to be efficiently phagocytosed such as *P. verrucosa* hyphae. Using NETs, multiple neutrophils can entrap particular microbes and microbial structures [8, 34], highlighting such NET-mediated killing as an important mechanism whereby neutrophils kill large pathogens.

Overall, our data provided novel evidence that *P. verrucosa* can induce NET formation by neutrophils. Given that extracellular killing appeared to be the main mode by which the hyphae of this fungus were killed in our studies, we speculate that extracellular NETs may be crucial to the in vivo control of this pathogen. Further work will be necessary to explore the in vivo relevance of NETs in this pathogenic context, but our data nonetheless underscore the importance of these mediators of innate immune responses to this infection type. Additional research into the relationship between NETs and *P. verrucosa* may aid in the development of novel antifungal agents.

## Data Availability

All data generated or analyzed during this study are included in this published article. The data used or analysed during the current study are available from the corresponding author on reasonable request.
